# Digital twins of the natural environment

**DOI:** 10.1016/j.patter.2021.100359

**Published:** 2021-10-08

**Authors:** Gordon S. Blair

**Affiliations:** 1 School of Computing and Communications, Lancaster University, Lancaster, UK

## Abstract

Digital twins emerged in the field of engineering but are now being applied in many areas of study. This article reflects on the enormous potential of digital twins of the natural environment and proposes an approach that builds on the massive legacy of process model understanding in this area combined with new insights from data understanding, including from AI/machine learning.

## Main text

### The ubiquitous “digital twin”

My favorite restaurant in Glasgow, the city of my birth, was the Ubiquitous Chip, with the name playfully poking fun at the ubiquity of the deep fat fryer in the cuisine of my homeland. It is another aspect of ubiquity that is holding my attention at the moment though—the ubiquitous “digital twin.” Digital twins are everywhere, and no matter where I turn, people are talking about the concept and how it is going to revolutionize our futures. Digital twins initially emerged in engineering to mean a digital or virtual representation of a physical artifact and one that is constantly updated to represent the current structure and behavior of that artifact. For example, imagine a digital twin for a motor engine: such a digital twin should represent the current running of that motor, including how all the various sub-components are operating and interacting, and such a twin can then be used, for example, to diagnose faults as or before they occur or to optimize the running of the engine for current conditions. Given the relatively closed world of engineering systems, it is quite easy to anticipate how such a digital twin can be constructed and also relatively straightforward to see the potential in this area (I say this with caution, though, as I am sure my friends in engineering will put me right about the complexities of the artifacts they construct). It is also, for me, quite easy to extrapolate from this and anticipate how digital twins can become key components at the heart of smart buildings or even smart cities. But what does it mean to develop digital twins related to different aspects of the natural environment? I have been to several very stimulating workshops and discussions on this topic recently, and this short article is my attempt to make sense of what I heard and what I am thinking.

### Why digital twins of the natural environment?

It is a curious feature of the literature and associated debate around digital twins that the emphases tend to be on *what* and *how*. I would argue that this is the wrong way round and it is really important to start with *why* digital twins can help in a given area. This is particularly important in considerations of the natural environment: significant investments are planned in this area,^1^^,^^2^ and it is crucial to think how digital twins are driven by demonstrable need related to the science and management of the natural environment. On reflecting on this question, I am convinced that there is a strong case to be made. First of all, the field of environmental science is undergoing a period of profound change. The natural environment is a highly complex system and one that is under unprecedented pressure from climate change, and in response to this, scientists are increasingly being asked to answer big questions in the face of this complexity and associated uncertainties. As a result, science is becoming more integrative, collaborative, and cross-disciplinary in nature and new tools are urgently required to support this new style of science. In parallel, there are new opportunities in environmental science due to the unprecedented availability of data from remote sensing, from instrumentation of the earth’s surface or indeed sub-surface, from citizen science, and from mining data available on the web. Again, though, this comes down to having new tools that help scientists make sense of this highly complex, rather messy data.^3^ This raises the key and pivotal question—*can digital twins fill this void* and offer up the tools that environmental science needs in response to the pressures to scale up the science and to properly enable a new, more data-driven style of investigation? This is a compelling argument, and given the urgency and importance of the science debates around climate change, flooding, drought, biodiversity loss, water and air quality, and food insecurity, is there a more important area to explore the concept of digital twins?

### But is this not just a model?

This is one reaction I often hear when digital twins are discussed in the environmental domain. This is a very valid question, especially given the long history of developing sophisticated models as key tools in the environmental sciences. And are these tools not digital twins? For me, the answer is “yes” and “no,” but with the evidence increasingly stacking up heavily on the “no” side. Digital twins are models, and environmental models offer sophisticated representations of underlying environmental phenomena. Many of these are process models that capture the current underlying scientific understanding encoded in mathematical models of the various underlying components and interactions. This is an impressive body of work, and it is a valid question to ask if these can be used as digital twins, perhaps enhanced with more integrated modeling to enable reasoning over more complete ecosystems. This argument falls down when you consider the narrative around data and the explosion of availability of data around the natural environment. Process models do not as yet take full advantage of this availability of “big data” around the natural environment (although data play an important tool in calibrating models). However, these data are crucial in helping us to have digital twins that are constantly updated to represent current observations and associated knowledge. This leads us to a consideration of big data techniques, stemming from data science and AI. There is less experience in applying such techniques in the environmental sciences, but this is quickly changing. So, can we construct a digital twin around big data and AI? Can we unravel the intricacies of the natural environment by applying machine learning and/or deep learning techniques to the resultant multi-dimensional data lakes? The answer to this question is much clearer to me—no. While big data is a key driver for digital twins, and data science has a crucial role in making sense of this data, to me it does not make sense to build a digital twin solely around AI. The killer argument for me is that process understanding is core to science, and science can only progress with deepening understanding of the associated processes and interactions. Process models are therefore core to digital twins of the natural environment, but only if they become more dynamic structures that evolve over time. I now see process modeling and data modeling as two points on a spectrum, and the key to digital twins is to identify the *sweet spot* where process understanding and data understanding can work together to best deepen our understanding and capture the dynamics and complexities of the phenomena being investigated.

### But is this not just data assimilation?

This is another question I often hear raised. Again, there is a strong tradition in many areas of environmental science to update model state dynamically so that the model fits with current observations, and this is a key technique in weather forecasting, for example. So, is this bringing together of data and process knowledge not just data assimilation? Again, this is a very valid question, but to me, it constrains thinking about digital twins too much, reducing the relationship between data and process understanding to one dimension—to improve model state based on current observation. This will be important in digital twin architectures, but there are many other dimensions to what is a rich, two-way relationship between data and process understanding. At this point, we enter unknown and largely unexplored territory, at least from my perspective. I see rich opportunity in this area: can we extract new meaning from available data using data science to extract new insights into extremes, changepoints, clusters, and correlations that can inform the science; are these observations meaningful, and if they are, do different process models accurately capture these behaviors and associated dynamics of the systems under observation; if not, can we amend not just the state but potentially also the structure of models or indeed the mix of models in the case of ensembles; are there useful flows in the opposite directions, for example, can process understanding or gaps/uncertainties in that understanding drive data capture and subsequent analyses? This is rich territory, and for me, this is the *intellectual heart* of a digital twin of the natural environment. Returning to my first question (“is this not just a model?”), at this point I am starting to see that this is actually becoming quite a step change in our modeling capabilities that sit at the heart of environmental science.

### What are the challenges?

This is not easy. Indeed, building a digital twin of some aspects of the natural environment represents a truly grand challenge and one that needs strong cross-disciplinary collaboration, bringing together environmental scientists with data sciences and computer scientists, among others. The challenges involved are plentiful, and I find it helpful to think of them in terms of a “jam sandwich” metaphor. First of all, there are three key “bread and butter” challenges that are necessary building blocks of any digital twin:

1. *Integration*: bringing the environmental assets together in one logical place, including both data assets and modeling assets (and my personal view is that virtual labs provide the right building block to achieve this, albeit with work to do to enhance virtual labs with the necessary infrastructure to support integrated data and models^4^).
2. *Interoperability*: allowing different assets to work together as part of a larger digital twin architecture, again also to include interoperable model components to support integrated modeling.
3. *Scalability*: to ensure that the necessary storage and processing capacity is available when it is needed, especially given the sizes of the challenges and the associated potentially very large datasets.

Although these look relatively straightforward, in practice they are demanding, especially the first two. Together, they also rely on a move to open data (and more generally open science) as an important underpinning for the necessary level of integration and interoperability. I am more confident in the scalability, given the engineering advances that underpin cloud computing, an important building block for digital twins. Overall, though, let us not underestimate the bread and butter challenges associated with digital twins; they remain significant issues in spite of much attention in the environmental sciences over the last decades.

Layered on top of that, we come to the “jam,” the value-added challenges that must be addressed to achieve the vision of digital twins. This is more open ended, but this list certainly includes:

1. *Data science and AI techniques for the natural environment*: there is a need for tailored data science and AI techniques that address the particular and arguably unique challenges of the natural environment (e.g., the data are complex and highly heterogeneous and exist at different temporal and spatial scales, and there is a need to reason about uncertainty across end-to-end pathways from data acquisition through to decision).^3^
2. *Process and data model integration*: as mentioned above, there is a need to fully understand the potentially synergistic relationship between data and process understanding and to derive software architectures where the associated models can work together, and indeed, this is a definitional aspect, to me, of a digital twin in this arena.
3. *Considerations of complexity*: As mentioned above, environmental systems, by their very nature, are highly complex and exhibit unexpected, emergent behavior. There is a need for modeling systems to better capture such complexity, including interactions, couplings, feedbacks, and dynamics in the system, and a subsequent need to look at complexity through new lenses, including input from the emergent area of complexity science.

Looking at the set of challenges, the complexity of building digital twins should not be underestimated. The term “moonshot” is overused these days, but perhaps digital twins represent a moonshot challenge for the environmental sciences. I am certainly of the opinion that to do this in a half-baked manner is equivalent to not doing it at all, as things will revert to more familiar approaches and science paradigms (*cf.* better process or data models) instead of really embracing the challenges.

### A final plea

I am excited about the prospect of seeing digital twins emerge for the natural environment. I am convinced there are opportunities to do something quite transformative *for the science*. I see risks, though; that we will rush toward building exemplars at the glamourous end of the spectrum; e.g., to build a new generation of earth system models. This would be exciting, but let’s not forget the range of scales that we operate at in the environmental sciences and indeed the important interconnections across scales. So, let us make space for global, national, regional, local, and hyper-local levels. And for me, some of the most exciting work will happen at the latter points on this scale, especially as we reason about place. This takes me back to recent work I did with Keith Beven and others as we revisited Beven’s concept of models of everywhere (more completely represented as models of everywhere, everything, and at all times).^5^ Is this not precisely what we seek as we contemplate digital twins related to place? But perhaps that is a different story.
